# Risedronate-Induced Chronic Drug Fever in a Case of Parkinson’s Disease

**DOI:** 10.7759/cureus.60117

**Published:** 2024-05-11

**Authors:** Yuji Higaki, Yuki Ito

**Affiliations:** 1 Department of Neurology, Yowa Hospital, Yonago, JPN; 2 Department of Psychiatry, Yowa Hospital, Yonago, JPN

**Keywords:** amino-bisphosphonates, parkinson’s disease, osteoporosis, c-reactive protein, chronic fever, risedronate, drug fever

## Abstract

We present an atypical case of risedronate-induced chronic fever in an 85-year-old woman with Parkinson’s disease, with a dosage regimen of 17.5 mg/week. Our patient had been administered an analgesic/antipyretic drug, acetaminophen, at a rate of 600 mg/day for treatment of a vertebral fracture that occurred relatively frequently, which might have masked the fever caused by risedronate. We noted two clinically significant indications. Firstly, blood test results do not necessarily show the cause of risedronate-induced fever, as white blood cell counts and C-reactive protein levels vary. A simple way to diagnose risedronate-induced fever is to suspend risedronate for a certain period and observe if the patient’s fever lowers. Secondly, in general, cases receiving polypharmacy tend to include an analgesic antipyretic agent, which may mask the drug-induced fever. Even in patients with Parkinson’s disease whose body temperature is generally unstable due to autonomic nerve system disorder, if they are administered risedronate and experience chronic fever of unknown cause, the possibility of drug fever may be considered. This study concludes that risedronate-induced chronic fever, as observed in our case, represents a rare phenomenon, and it may be necessary to reconsider treatment methods for osteoporosis.

## Introduction

Risedronate is a potent inhibitor of bone resorption that has been shown in several controlled studies to significantly reduce the risk of fragility fractures [1]. Although rare, side effects may include gastrointestinal disturbances and acute phase response (APR), such as fever, chills, and myalgia, which occur within five days of the first dose and have been reported to last up to seven days [1]. Shah et al. reported the case of a 76-year-old woman who was admitted to a tertiary care hospital six times over a six-month period with fever and abdominal symptoms that were consequently attributed to risedronate after various diagnostic tests [2].

In our case, an 85-year-old female patient with Parkinson's disease (PD) experienced chronic fever while receiving risedronate (17.5 mg) once weekly for nearly 16 years to manage osteoporosis. It has been known that bisphosphonates, including risedronate, can induce acute fever lasting only a few days after administration. To the best of our knowledge, risedronate-induced chronic fever has been rarely reported. In this report, we present an atypical case of risedronate-induced chronic fever.

## Case presentation

An 85-year-old female patient had a clinical history of depression, PD, multiple compression fractures of the thoracolumbar spine, and cataracts in both eyes that had been surgically treated. In 1999, at the age of 61, the patient began experiencing tremors while resting. Despite consulting three clinics, the cause was not determined. She was diagnosed with depression and started receiving treatment; however, her symptoms persisted. In June 2007, at the age of 69, based on imaging examinations and physical symptoms, including right-side-dominant tremors while resting, muscular rigidity, and postural instability, she was eventually diagnosed with PD by a neurologist at University Hospital B. Thus, a combination of levodopa and carbidopa (200 mg/day) was orally administered, and outpatient follow-up observation was initiated. In November 2007, the patient developed a minor lumbar compression fracture and was treated conservatively. In March 2011, at the age of 73, she had suffered another lumbar compression fracture.

She had been suffering from cervical dyskinesia since 2007, and weekly oral administration of risedronate was initiated. Since then, her husband noticed her temperature was approximately 1℃ higher than her normal body temperature of around 35.8℃. In October 2020, at the age of 82, she suffered a thoracic compression fracture, and in January 2021, she underwent surgery for a left femoral neck fracture caused by a fall. Subsequently, the patient developed hallucinatory and delusional symptoms. In August 2023, she repeatedly began to engage in self-harming behaviors. In late August 2023, she was admitted to our hospital due to hallucinations and suicide attempts.

Clinical findings upon admission

Upon admission to our hospital, her body temperature was 37.4°C, her blood pressure was 99/69 mmHg, and her heart rate was 84 bpm. Tachypnea was not observed, and her indoor blood oxygen saturation level was 95%. She did not experience headaches, nausea, or pain in the muscles of her neck, shoulders, or upper arms. She had no joint swelling, tenderness, or redness. She had no respiratory symptoms, abdominal or urinary pain, skin rash, or palpable lymph nodes on her extremities. She had no heart murmurs. None of the patient’s relatives had been diagnosed with PD. Blood tests revealed normal white blood cell counts, C-reactive protein (CRP), transaminase, creatinine phosphokinase, urinalysis, and thyroid function. The antinuclear antibody and rheumatoid factor levels were normal (Table 1). No abnormalities were observed on chest radiography.

**Table 1:** Blood and urine test results. ANA: antinuclear antibody; RF: rheumatoid factor; ALP: alkaline phosphatase; IFCC: International Federation of Clinical Chemistry and Laboratory Medicine; ALT: alanine aminotransferase; AST: aspartate aminotransferase; CPK: creatine phosphokinase; γGTP: γ-glutamyl transpeptidase; LDH: lactate dehydrogenase; WBC: white blood cells; CRP: C-reactive protein.

Year 2023	August 24	September 13	September 25	October 16	November 27	Normal range
AST	14	14	13	16	13	13-30 IU/l
ALT	7	11	9	17	5	7-23 IU/l
LDH	169	-	154	159	199	124-222 IU/l
ALP (IFCC)	67	-	86	90	85	38-113 U/L
γGTP	14	17	11	15	13	9-32 IU/l
CPK	28	-	35	-	65	41-153 IU/l
Alb	3.9	-	3.7	4.1	4.2	4.1-5.1 g/dl
Ca	9.1	-	8.5	-	9.1	8.8-10.1 mg/dl
Hb	11.3	12.6	11.2	12.2	12	12-16 g/dl
WBC	4300	6500	5300	5500	8100	4200-7700
CRP	0.02	0.01	0.01	0.05	0.02	Equal or less than 0.3 mg/dl
TSH	0.71	-	-	-	-	-
Free T4	1.45	-	-	-	-	-
Urinalysis						
Bacteria	(-)	-	(-)	-	-	(-)
ANA	-	-	-	-	Less than 40	0-40
RF	-	-	-	-	Less than 4	0-15 IU/ml
25(OH)VD	-	-	-	-	10.2	30.0-100.0 ng/ml

**Figure 1:**
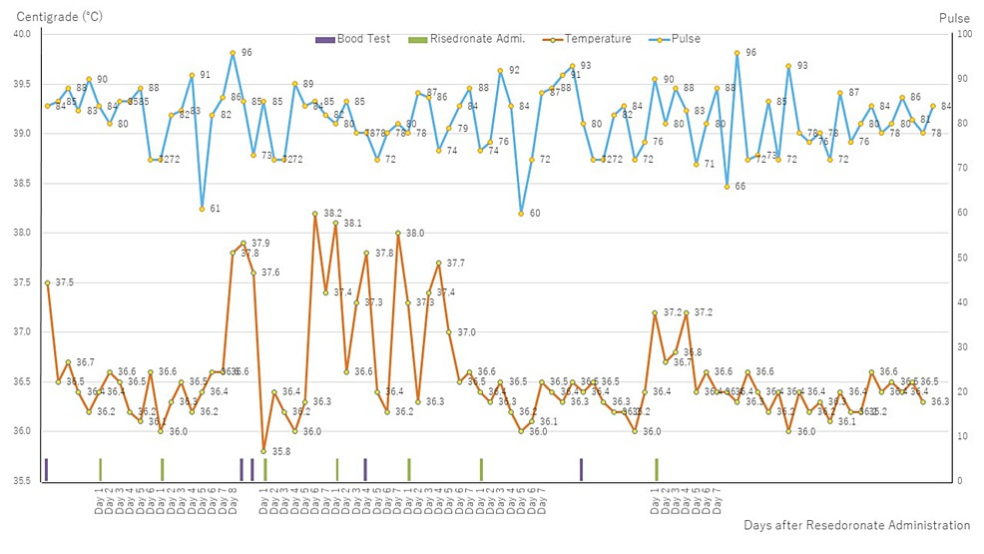
Body temperature and pulse from admission to discharge.

After admission, she showed intermittent fever ranging from 36°C to 38°C, although her normal body temperature had been around 36.0 to 36.5°C (Figure 1). She had been receiving 14 different medications at the time of admission; therefore, over a period of one month, we attempted to reduce the number of medications that were likely to cause the side effects of hallucinations and delusions. Consequently, the number of prescribed medications was reduced to seven (Table 2). Subsequently, she calmed down, and her hallucinations and delusions significantly improved.

**Table 2:** Reduced or changed drugs over a period of one month.

Prescribed drugs	August 24, 2023	September 26, 2023	Notes (main purpose and effects)
Acetaminophen	Tablet 200 mg × three times/day (after meal)	-	For treatment of inflammation
Mecobalamin	Tablet 500 μg × three times/day (after meal)	-	For treatment of numbness
Rosuvastatin	Tablet 2.5 mg/day (after breakfast)	-	To reduce cholesterol levels (Crestor®)
Mirabegron	Tablet 25 mg/day (after breakfast)	-	For treatment of overactive bladder
Magnesium oxide	Tablet 500 mg × two times/day (after breakfast and dinner)	-	For treatment of constipation
Mosapride	Tablet 5 mg × three times/day (after meal)	-	For treatment of gastric mucosal injury
Rabeprazole	Tablet 10 mg/day (after breakfast)	-	For treatment of esophagitis regurgitica (Pariet®)
Risedronate	Tablet 17.5 mg/week	Tablet: 17.5 mg/week	For treatment of osteoporosis
Zonisamide	Tablet 50 mg/day (after breakfast)	-	For treatment of Parkinson's disease
Stalevo L100® (Levodopa 100 mg, Carbidopa 10 mg, Entacapone 100 mg)	Tablet: Stalevo L100® × three times/day (after meal)	-	For treatment of Parkinson's disease (Entacapone may induce hallucination and paranoia as adverse reactions)
Dopacol L100® (Levodopa 100 mg, Carbidopa 10 mg)	Tablet: Dopacol L100®/day (upon waking up)	Tablet: Dopacol L100® × three times/day (after meal)	For treatment of Parkinson's disease (substitute for Stalevo L100, eliminating entacapone)
Yokukansan	Granules: Yokukansan 2.5 g × three times/day (after meal)	-	Herbal medicine to treat the unsettled mind
Daikenchuto	Granules: Daikenchuto 2.5 g × three times/day (after meal)	-	Herbal medicine to treat abdominal distention
Donepezil	Tablet: 3 mg/day (after breakfast)	-	For treatment of Alzheimer's disease
Trihexyphenidyl	-	Tablet: 2 mg/day (after breakfast)	To reduce shaking (Artene®)
Amlodipine	-	Tablet: 5 mg/day (before sleep)	To reduce blood pressure in the night
Rhubarb licorice soup	-	Granules: 2.5g × three times/day (after meal)	Herbal medicine to treat constipation
Folic acid	-	Tablet: 5 mg/day (after breakfast)	Vitamin to improve folic acid level in blood
Pora Prezinc	-	Tablet: 75 mg/day (after breakfast)	Vitamin to improve zinc level in blood
Total number of drugs	14	Seven	-

However, although five blood tests showed that her white blood cell count and CRP were almost within the normal range, her fever persisted. A literature search revealed a case of recurrent fever caused by risedronate treatment [2]. Therefore, risedronate was suspended for 16 days, starting on October 7th. Consequently, the fever subsided for over 16 days. Then, as a challenge test, on October 23rd, 17.5 mg of risedronate was orally administered; subsequently, a mild fever of 37.2°C was observed in the evening of the same day, which lasted for three days. Subsequently, we again suspended risedronate and observed the patient for a month, after which she did not experience any fever. Therefore, the patient was diagnosed with risedronate-induced chronic fever. According to the adverse drug reaction (ADR) algorithm reported by Naranjo et al. [3], an ADR score of nine was categorized as a definite ADR. Later, in November 2023, she was discharged from the hospital and followed up monthly at an outpatient clinic. As of mid-January 2024 (three months after suspending risedronate), her body temperature has been staying in her normal range, not exceeding 37°C, and her vital signs and mental state have been stable.

Written consent to publish the contents of this case report was obtained from the patient in accordance with the Declaration of Helsinki.

## Discussion

Our case differs from those of previous studies regarding drug-induced fever for the following two reasons: (1) Although acute phase response (APR) is a known phenomenon of risedronate that occurs only after the first administration and lasts for a maximum of seven days [1], in this case, the patient’s fever persisted beyond that time frame. According to the drug information sheet of risedronate, APR tends to occur after the second administration and may occur repeatedly. The fever of our case might be due to repetitively occurred APRs. Another notable thing is that the C-reactive protein level of our case was within the normal range in all four measurements (Table 1). (2) Our case had been administered acetaminophen at the rate of 600 mg/day to relieve continuous pain due to a vertebral fracture that occurred relatively frequently, which might have masked the fever caused by risedronate. In our case, after suspending three antipsychotic drugs (zonisamide, entacapone, and donepezil), hallucinosis and delusion disappeared. Neurologic manifestation may be reduced by limiting the number of drugs, which would most likely lead to a more stable mentality, even in cases of Parkinson’s disease.

Regarding no. 1 above, Nuti et al. stated in their review that APR is occasionally caused by oral administration of risedronate for the treatment of osteoporosis, and the phenomenon continued for a maximum of seven days after the initial administration. We considered our patient’s fever chronic because she was admitted to our hospital in an emergency, not primarily for her fever, although she had a mild fever of 37.5°C on admission. Though her COVID-19 polymerase chain reaction (PCR) test results were negative, she had been prescribed 14 drugs (Table 2). Therefore, over a period of one month, we gradually reduced the number of drugs to seven, but her fever persisted (Table 2). Then, we searched the literature and found a case report by Shah et al., who described risedronate-induced drug fever in a 76-year-old woman who was admitted six times for abdominal pain. Therefore, we decided to discontinue risedronate on the 43rd day after the initial admission. No fever was observed for 16 days while risedronate was suspended, and the patient’s condition improved, allowing for sufficient food intake. Subsequently, her body weight increased from 28.4 kg to 37.8 kg in two and a half months. Therefore, we hypothesized that risedronate was the most likely cause of the chronic fever. To confirm this hypothesis, we conducted a risedronate challenge test on the morning of the 60th day after admission. Afterward, her temperature increased from 36.4 to 37.2°C in the evening, and a mild fever lasted for three days. Therefore, we considered that the cause of the chronic fever was probably due to risedronate. Based on these findings, we diagnosed the patient with risedronate-induced drug fever.

Regarding our patient’s normal CRP levels even when her body temperature was elevated, Olson et al. reported a comparison of three amino-bisphosphonate (BP) drugs: variations of cytokines, kinetics of CRP, and immunocytes with the occurrence of fever [4]. They stated that the use of ibandronate will not affect CRP levels, irrespective of the presence of fever, whereas zoledronate will elevate CRP levels, irrespective of the presence of fever. Individual differences in susceptibility to the administered drug may explain the presence or absence of a drug-induced fever. Patel et al. pointed out that normal values do not preclude the diagnosis of drug-induced fever, and that no other laboratory tests are consistently useful for diagnosis [5].

Regarding no. 2 above, polypharmacy in elderly patients tends to mask certain adverse reactions caused by concomitant use of other drugs. Giovannini et al. [6] investigated the ratio of hospitalization in 165 patients with Parkinson’s disease, comparing the total number of prescribed drugs: one to three drugs, four to six drugs, and over seven drugs. Their Kaplan-Meier curve graph showed a smaller number of prescribed drugs tended to indicate a larger number of non-hospitalized patients, while in cases with four or more prescribed drugs, the hospitalization rate was over 50% [6]. Even when excluding drugs not related to antipsychotic effects, this tendency was the same, suggesting a greater number of drugs for the treatment of Parkinson’s disease may also be the cause of a high hospitalization ratio [6]. Potentially, in clinical practice, there may be more patients with chronic fever caused by amino-BP agents, including risedronate. One of the reasons for noticing drug-induced fever in our case was frequent examinations of physical conditions after the patient was hospitalized. The chronic fever was not much examined, while the patient had been receiving treatment at home. Further investigation is desired to verify our hypothesis regarding the timing of drug-induced fever. In addition, the occurrence of fever with atypical blood test results of low C-reactive protein levels indicates the need to clarify pathophysiological mechanisms for such reactions.

## Conclusions

Even in patients with Parkinson’s disease whose body temperature is generally unstable due to autonomic nerve system disorder, if they are administered risedronate and experience chronic fever of unknown cause, the possibility of drug fever may be considered. This may be confirmed by suspending the use of risedronate. In general, one of the problems of polypharmacy in elderly patients is that certain adverse reactions might be masked by the concomitant use of other drugs. This study concludes that risedronate-induced chronic fever, as observed in this case, represents a rare phenomenon, and it may be necessary to carefully reconsider treatment methods for osteoporosis.
